# Differential drug resistance acquisition to doxorubicin and paclitaxel in breast cancer cells

**DOI:** 10.1186/s12935-014-0142-4

**Published:** 2014-12-21

**Authors:** Feifei Xu, Fengliang Wang, Ting Yang, Yuan Sheng, Ting Zhong, Yun Chen

**Affiliations:** School of Pharmacy, Nanjing Medical University, 818 Tian Yuan East Road, Nanjing, 211166 China; Nanjing Maternity and Child Health Care Hospital, Nanjing, 210004 China

**Keywords:** Multi-drug Resistance Acquisition (MDR), P-glycoprotein (P-gp), Drug-specific, Doxorubicin (DOX), Paclitaxel (PTX)

## Abstract

**Background:**

Several signal transduction pathways have been reported being involved in the acquisition of P-glycoprotein (P-gp) mediated multi-drug resistance (MDR) upon exposure to anti-cancer drugs, whereas there is evidence indicating that the expression and activity of P-gp were not equally or even reversely modulated by different drugs.

**Methods:**

To further illustrate this drug-specific effect, possible mechanisms that enable breast cancer cells MCF-7 to acquire MDR to either paclitaxel (PTX) or doxorubicin (DOX) were investigated in a time-dependent manner.

**Results:**

The results suggested that at least two pathways participated in this process. One was the short and transient activation of NF-κB, the second one was the relatively prolonged induction of PXR. Both PXR and NF-κB pathways took part in the PTX drug resistance acquisition, whereas DOX did not exert a significant effect on the PXR-mediated induction of P-gp. Furthermore, the property of NF-κB activation shared by DOX and PTX was not identical. An attempt made in the present study demonstrated that the acquired resistance to DOX was *via* or partially *via* NF-κB activation but not its upstream receptor TLR4, while PTX can induce the drug resistance *via* TLR4-NF-κB pathway.

**Conclusions:**

To our knowledge, this report is among the first to directly compare the time dependence of NF-κB and PXR pathways. The current study provides useful insight into the distinct ability of DOX and PTX to induce P-gp mediated MDR in breast cancer. Different strategies may be required to circumvent MDR in the presence of different anti-cancer drugs.

**Electronic supplementary material:**

The online version of this article (doi:10.1186/s12935-014-0142-4) contains supplementary material, which is available to authorized users.

## Background

Chemotherapeutics are an effective treatment against tumors. However, the efficacy of the treatment may be impeded by multi-drug resistance (MDR), a phenomenon that consists in the development of resistance by the tumor [1]. In the case of breast cancer, initially responsive tumors frequently relapse and acquire resistance to a broad spectrum of drugs after the initial sensitivity to cytotoxic agents [2]. Among the recognized mechanisms underlying the MDR, the efflux of the drugs by ATP binding cassette (ABC) transporters in the membrane compartments was the most concerned one [3]. Even though this mechanism has not been unveiled completely, avoiding the appearance of drug resistance and modulating the MDR have been a great challenge to cancer therapy in clinic and laboratory.

Among the ABC transporters, P-glycoprotein (P-gp) is the first described and most extensively studied one [4]. This transporter protein is encoded by the gene multi-drug resistance 1 (MDR1) in human and is expressed widely in epithelial cells of many organs [5]. Its expression can be altered by a variety of xenobiotics, thus affecting its transport activity [6]. As typically and commonly used cytotoxic drugs in the treatment of breast cancer, paclitaxel (PTX, a microtubule-stabilizing drug) and doxorubicin (DOX, a DNA damaging drug) can both enhance the P-gp expression and induce MDR, resulting in the failure of chemotherapy [7]. However, cDNA microarray analysis provided the evidence that isogenic PTX- and DOX-resistant breast cancer cells indicated distinct drug-specific genetic signatures of resistance that accompanied the establishment of PTX or DOX resistance [8]. Differences in the capacity of drugs to induce cross-resistance to each other have been also reported for DOX and PTX [7]. The basis for this drug-specific effect in the process of drug resistance acquisition is so far unclear. Currently, of particular importance is to know the extent to which the transporter activity is path-physiologically modulated.

To date, a number of studies have described that activation of nuclear factor-kappa B (NF-κB) leads to the alternation of P-gp activity. However, the results of these studies are inconclusive. Most of them suggested that P-gp was up-regulated by NF-κB activation [9,10], while others demonstrated a down-regulation effect [11]. In addition to NF-κB, another inducer associated with the P-gp expression and activity is pregnane X receptor (PXR). As an orphan nuclear receptor known for its activation by structurally diverse compounds, PXR is implicated as a novel regulator of MDR in cancers [12]. Even though a variety of xenobiotics and endogenous ligands have been reported as the activators of PXR and NF-κB [13,14], whether PXR and NF-κB pathways cause the difference of resistance acquisition to DOX and PTX have not been explored yet.

In this study, we investigated the impact of DOX and PTX on the expression and transport activity of P-gp in breast cancer cells MCF-7. The potential modulation of drugs on the NF-κB and PXR-mediated P-gp induction were evaluated. Since toll-like receptor 4 (TLR4) signaling pathway generally culminates in the activation of NF-κB [15], the role of TLR4 was also investigated. The ultimate aim of this study is to provide further insight into the P-gp mediated MDR acquisition and enhance the efficacy of breast cancer treatment in the presence of different chemotherapy drugs.

## Materials and methods

### Materials and reagents

PTX was purchased from Hisun Pharmaceutical Co., Ltd. (Zhejiang, China) and DOX was from Sigma-Aldrich (St. Louis, MO, USA). Rhodamine 123 (Rho-123) and Bay 11–7082 were both supplied by Sigma-Aldrich (St. Louis, MO, USA). Triton X-100 and phenylmethanesulfonyl fluoride (PMSF) were from Generay Biotech Co., Ltd. (Shanghai, China). Deoxycholate was from Biosharp (Huaibei, Anhui, China). Penicillin was obtained from CSPC Zhongnuo Pharmaceutical Co., Ltd (Shijiazhuang, China). Streptomycin was obtained from Merro Pharmaceutical Co., Ltd (Dalian, China).

### Cell culture and drug treatment

Human breast cancer cell line MCF-7 was purchased from ATCC (Rockville, MD, USA) and its corresponding DOX-resistant cells (MCF-7/DOX) were obtained from KeyGEN BioTech (Nanjing, China). PTX-resistant cells (MCF-7/PTX) was generously donated by Dr. Sun [16]. MCF-7/DOX cells were cultured in RPMI 1640 media (with L-glutamine and sodium pyruvate) supplemented with 10% fetal bovine serum (Thermo Scientific Hyclone, Utah, USA) at 37°C and 5% CO_2_. MCF-7 and MCF-7/PTX cells were maintained routinely in DMEM media supplemented with 10% fetal bovine serum, 100 U/mL penicillin and 100 μg/mL streptomycin.

### Reverse transcriptase (RT)-PCR and real-time quantitative RT-PCR (qRT-PCR)

Total RNA was isolated from cells using RNAiso Plus reagent (Takara, Dalian, China) according to the manufacture’s instructions and quantified using UV absorbance spectroscopy. For single-strand cDNA synthesis, 1 μg total RNA was reverse transcribed in a 10 μL reaction using the 5 × primescript RT Master Mix (Takara, Dalian, China) at 37°C for 15 min. The expression profiles of MDR1 that encodes P-gp in MCF-7, MCF-7/DOX and MCF-7/PTX cells were evaluated using RT-PCR with the MJ Mini Personal Thermal Cycler (Bio-Rad Laboratories, Richmond, USA). Details of primers and predicted band sizes of PCR products are shown in Additional file 1: Table S1. The amplified products were run on 3% Gel-red (Biotium, CA, USA) stained agarose gel and visualized under UV light.

qRT-PCR for PXR and MDR1 was done using an Applied Biosystems 7500 real-time PCR system (Applied Biosystems, CA, USA). The total reaction volume was 20 μL containing 2 × SYBR Green PCR master mix (Takara, Dalian, China), 200 nmol/L of each primer and 2 μL cDNA as template. After two initial steps of 50°C for 2 min and 95°C for 6 min, the PCR reaction was run 40 cycles of 95°C for 15 s and 60°C for 1 min. Melt cure analyses were done for all samples and only sharp melting points were observed, indicating a specific signal and no primer dimmers or mispriming. The Ct value is defined as the fractional cycle number at which the fluorescence passes the fixed threshold. Quantification of PXR and MDR1 mRNA for each sample was calculated by correcting for against the Ct level of GAPDH. The data are normalized to controls (untreated cells) and presented as mean data (± S.D.) of three different experiments.

### Measurement of P-gp activity in MCF-7 Cells

P-gp activity in MCF-7 cells was assessed based on Rho-123 uptake. Rho-123 is a typical substrate of P-gp, and a decrease or increase in Rho-123 uptake by cells reflects the enhancement or suppression of P-gp function, respectively. Briefly, cells were seeded onto 6-well plates and pretreated with drugs, then incubated with 2.5 μM Rho-123 in the dark at 37°C for 60 min. After washed twice with ice-cold PBS, cells were harvested in 400 μL of 1% (v/v) Triton X-100 in PBS. Then, the whole cell lysate was centrifuged at 13,000 rpm for 10 min at 4°C. The amount of Rho-123 was determined using the 96-well fluorescence plate reader with excitation/emission wavelengths at 510/536 nm using EnSpire 2300 (PerkinElmer, Waltham, MA, USA). The total protein concentration was measured by the BCA protein assay kit (Pierce, Rockford, USA) according to the manufacturer’s protocol. The amount of intracellular Rho-123 was normalized to the total protein concentration. The ratio of these data in the absence or presence of drugs/inhibitors is indicative for the activity of P-gp. The data are presented as mean data (± S.D.) of three different experiments. In this study, the initially selected concentrations of PTX were 1, 2.5, 5, 10, 25, 50 nM and those of DOX were 10, 25, 50, 100, 250, 500 nM. Then, the cells were pretreated with PTX and DOX at the concentrations having the maximal effect.

### Protein extraction and Western blotting

The cells were washed twice with ice-cold PBS and lysed in RIPA lysis buffer (Beyotime, Shanghai, China). After incubated on ice for 40 min, the whole cell lysate was centrifuged at 12,000 rpm to remove cellular debris. Cytosolic and nuclear proteins were isolated using the NE-PER nuclear and cytoplasmic extraction kit (Pierce, Rockford, USA) according to the manufacturer’s protocol. 50 μg of total protein was resolved by 4-12% SDS-PAGE, unless stated otherwise, and transferred onto a polyvinylidenefluoride (PVDF) membrane (Millipore, Billerica, MA, USA). Western blot analysis was performed using primary antibodies for PXR (1:1000, Biolegend, CA, USA), NF-κB p65 (1:500, Cell signaling, Beverly, MA, USA), P-gp (1:200, Abcam, Cambridge, UK) GAPDH, Actin (1:2000, Bioworld, Nanjing, China) and Anti-TATA binding protein (TBP) antibody (1:500, Proteintech, Chicago, IL, USA), followed by HRP-conjugated goat anti-mouse/rabbit IgG (1:5000, MultiSciences Biotech. Co., Ltd., Hangzhou, China). The proteins were then detected using enhanced chemiluminescence reagent (Pierce, Rockford, USA) according to the manufacturer’s protocol.

### Analysis of NF-κB p65 translocation using confocal microscopy

MCF-7 cells were grown on confocal petri dish and treated with two drugs for 24 h. Then, the cells were washed with PBS, fixed with 4% paraformaldehyde and permeabilized with PBST (0.2% Triton X-100 in PBS) solution for the entrance of NF-κB p65 antibody. The cells were blocked with 5% BSA in PBS, followed by the rabbit monoclonal NF-κB p65 antibody (1:25, Cell signaling, Beverly, USA) prepared in 3% BSA in PBS overnight. After washing three times with PBS, Dylight 488 Affinipure goat anti-rabbit IgG (H + L) (1:200, EarthOx, California, USA) secondary antibody was added and incubated at room temperature for 1 h. Then DAPI stain (10 μg/ml) for nuclei was added and images were acquired at room temperature using a LSM700 confocal microscope with a C-Apo × 40 water immersion lens (Carl Zeiss, Germany).

### Cellular modulation of PXR expression with small interfering RNA (siRNA)

The siRNA sequence targeting human PXR gene and the negative control that consisted of a non-complementary sequence were developed by GenePharma (Shanghai, China). Three different sequences were evaluated first. The PXR siRNA with the following sense and anti-sense sequences was chosen: 5′-GAUGGACGCUCAGAUGAAATT-3′ (sense) and 5′-UUUCAUCUGAGCGUC- CAUCTT-3′ (anti-sense). Cells were grown to approximately 80% confluence in 6-well plates and transfected with 20 pM PXR siRNA using Lipofectamine 2000 (Invitrogen, Breda, Netherlands). The cells were incubated at 37°C with 5% CO_2_ for subsequent analysis.

### Inhibition of TLR4 and NF-κB pathways

BAY 11–7082, which can block the phosphorylation of IκB and decrease the expression of adhesion molecules, was used as an NF-κB inhibitor [17]. MCF-7 cells were first grown in 6-well plates and incubated for 48 h, prior to the incubation with 5 μM BAY 11–7082 for 2 h, followed by PTX and DOX treatment. To measure the function of TLR4, which is the upstream signal transduction of NF-κB activation, TLR4 was blocked by a pretreatment with 2 μg of the anti-human TLR4-blocking antibody (Hycult Biotech, Holland).

### Data analysis

Mann–Whitney test was used to analyze the difference between two groups. Data were presented as mean ± S.D. The general acceptance level of significance was p < 0.05.

## Results

### P-gp and MDR1 mRNA expression in cells

**Figure 1:**
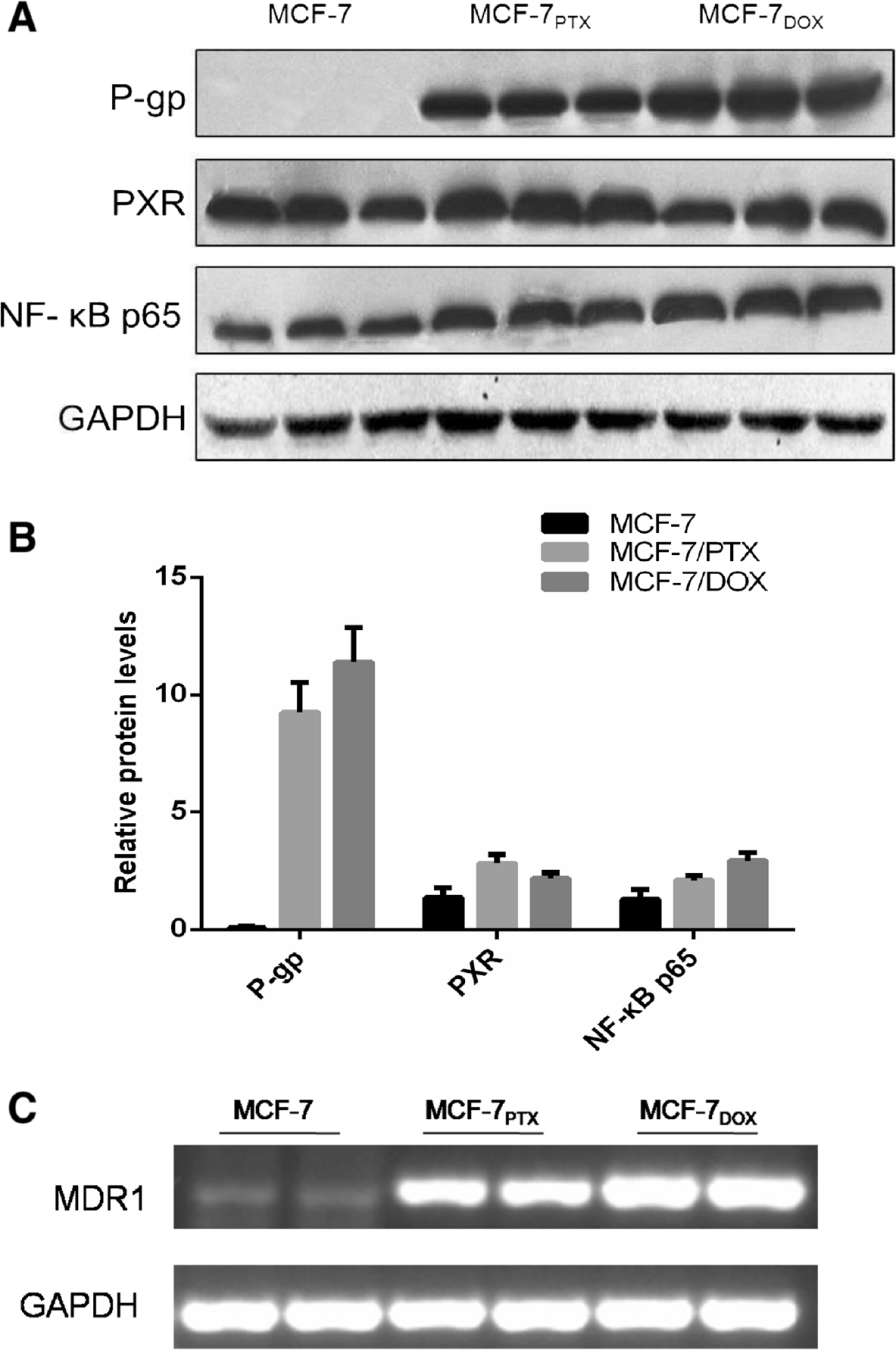
**Representative Western blotting (A) and quantitative analysis (B) of P-gp, PXR and NF-κB p65, and mRNA of MDR1 determined by RT-PCR (C).** GAPDH was used as the internal control.

Three MCF-7 cell lines were first phenotyped for P-gp expression using Western blotting. Consistent with previous studies [18,19], a well-defined band at approximately 170 kDa was detectable in MCF-7/DOX and MCF-7/PTX cells compared to their parental MCF-7 cells (Figure 1A and B). In addition, mRNA expression of P-gp was determined. As expected, similar result was obtained. MCF-7 cells expressed a low level of MDR1 mRNA, barely detectable using RT-PCR assay (Figure 1C). Up-regulation of MDR1 mRNA was observed in drug-resistant cells. Notably, the observed differences between Western blotting and RT-PCR results were consistent with the previous studies that the concordance between gene and protein expression findings usually ranged from poor to moderate [20]. Interestingly, both approaches indicated a difference in the increase of P-gp level between MCF-7/DOX and MCF-7/PTX cells. The enhancement of P-gp in MCF-7/DOX cells was ~1.7 fold greater than that in MCF-7/PTX cells.

### PXR and NF-κB expression in cells

Both PXR and NF-κB are highly likely responsible for the modulation of P-gp expression and activity. Thus, the expression PXR and NF-κB p65 in MCF-7/PTX and MCF-7/DOX cells were first conducted. Since activation of NF-κB is associated with nuclear translocation of the p65 component of the complex, quantification of nuclear p65 can be used as a parameter for NF-κB activation [21]. As shown in Figure 1A, MCF-7/DOX cells have a higher enhancement of NF-κB/p65, even though an increase was obtained in both MCF-7/DOX and MCF-7/PTX cells. On the other hand, MCF-7/PTX cells have a ~1.3-fold higher expression of PXR than its parental MCF-7 cells, whereas the difference of this receptor between MCF-7/DOX and MCF-7 cells was not easily distinguishable.

### Impact of DOX and PTX on P-gp expression and activity in short-term and long-term

**Figure 2:**
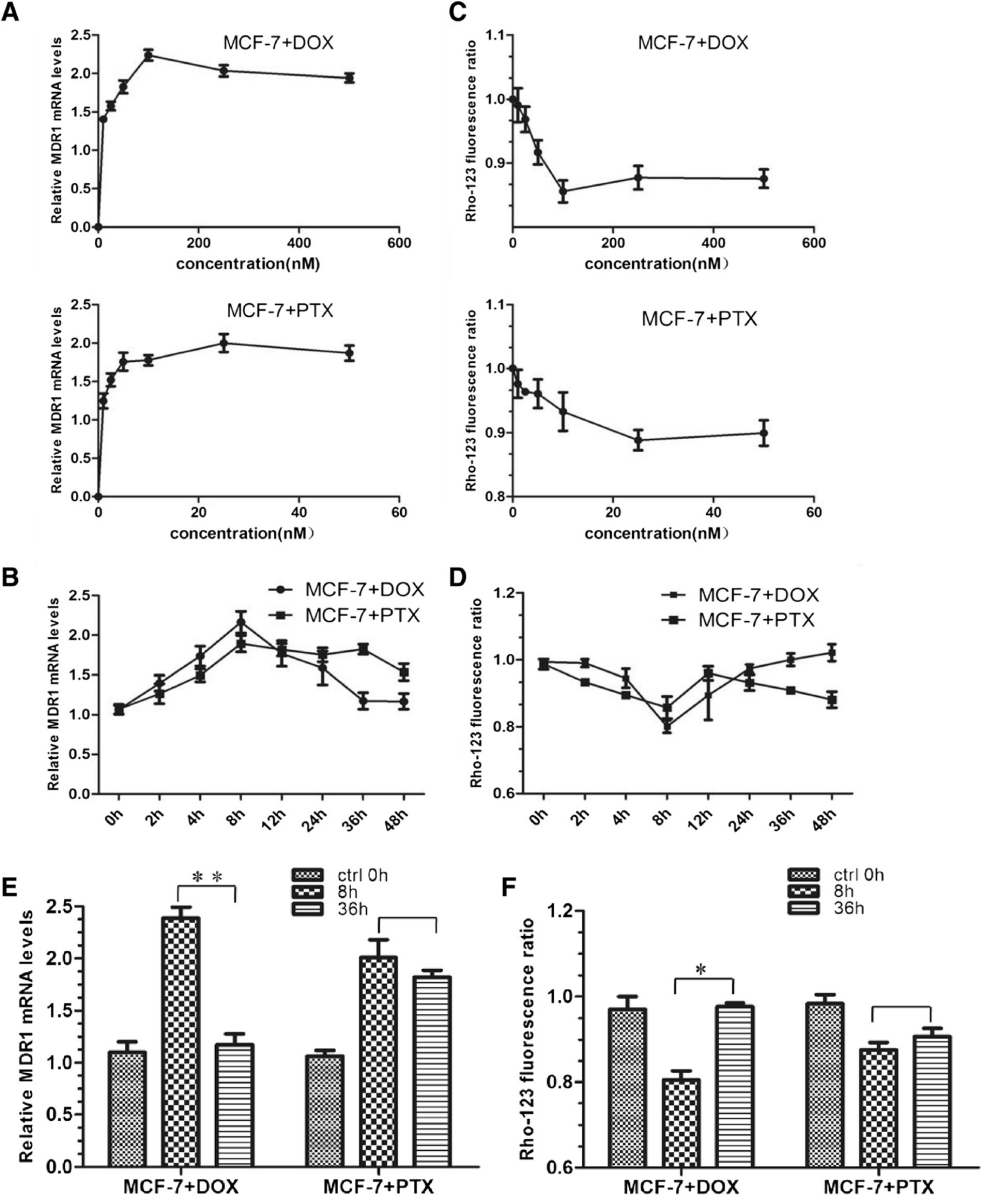
**The impact of DOX and PTX on P-gp expression and activity.** **(A)** The concentration-dependent effect of DOX and PTX on mRNA level of MDR1. The cells were treated with drugs for 12 h. **(B)** The time-dependent effect of drugs on mRNA levels of MDR1 with 100 nM DOX/25 nM PTX treatment. **(C)** Rho-123 fluorescence ratio in MCF-7 cells with DOX and PTX treatments at different concentrations. **(D)** Rho-123 fluorescence ratio in MCF-7 cells with 100 nM DOX/25 nM PTX treatment for 0, 2, 4, 8, 12, 24, 36, 48 h. **(E)** MDR1 mRNA levels in MCF-7 cells with 8 h/36 h DOX and PTX treatment. *, *p* < 0.05; **, *p* < 0.01 (n = 3). **(F)** Rho-123 fluorescence ratio in MCF-7 cells with 8 h/36 h DOX and PTX treatment. *, *p* < 0.05 (n = 3).

To closely monitor the PXR and NF-κB changes in the development of resistance to DOX and PTX, MCF-7 cells were induced by these drugs in a time course here. The first step in the experimental design is the selection of DOX and PTX concentration for the treatment of cells. Thus, an investigation was performed to evaluate the P-gp expression of MCF-7 cells as function of PTX and DOX concentrations, respectively. The selected concentrations of PTX were 1, 2.5, 5, 10, 25, 50 nM and those of DOX were 10, 25, 50, 100, 250, 500 nM, following their previously reported IC_50_ values [7]. The results suggested that the maximal effect of drugs were observed at DOX 100 nM and PTX 25 nM (Figure 2A and C) with 12 h treatment time. Therefore, these concentrations were used in the following experiments.

The cells were subsequently analyzed for mRNA levels of MDR1 after different drug treatment lengths. As a result, P-gp induction was an early event in the process of drug resistance acquisition (Figure 2B). After a comparison at individual time points, we found that the increase in the level of MDR1 mRNA after exposure to DOX and PTX reached 2.39 and 2.01 folds at 8 h, suggesting a transcriptional regulation of P-gp by these drugs (Figure 2E). A higher enhancement was achieved by DOX. However, this up-regulation was down-regulated following a more prolonged exposure (e.g., 36 h) to PTX (1.82 fold), whereas this alternation was not observed for DOX (1.17 fold, P > 0.05) (Figure 2E).

Meanwhile, P-gp transport activity was also analyzed. We exposed the cells to the fluorescent P-gp substrate Rho-123 and measured P-gp activity by comparing the intracellular accumulation of Rho-123 [22,23]. The concentration and time points were the same as those used in the qRT-PCR assay (Figure 2C-D). In agreement with protein level, intracellular Rho-123 significantly decreased in 8 h after drug exposure (80.1% for DOX and 87.5% for PTX), indicative of a declined P-gp function (Figure 2F). In a long-term exposure, a similar phenomenon to the mRNA result was also observed that the function of P-gp was resumed in DOX not PTX treated cells.

### Activation of NF-κB pathway

NF-κB is a transcription factor that induces the expression of genes involved in many biological responses including inflammation, cell proliferation and survival [24]. The majority of NF-κB exists as a heterodimer of p65/p50 proteins sequestered in the cytosol and bound to inhibitory IκB proteins [25]. Stimulation of cells can activate IκB kinases that phosphorylate IκB and result in ubiquitination and proteosomal degradation of IκB. Then the free p65/p50 complex enters the nucleus and initiates transcription of downstream effector genes [24]. Thus, the appearance of p65 in the nuclear extracts of cells can be a sign of NF-κB activation.

**Figure 3:**
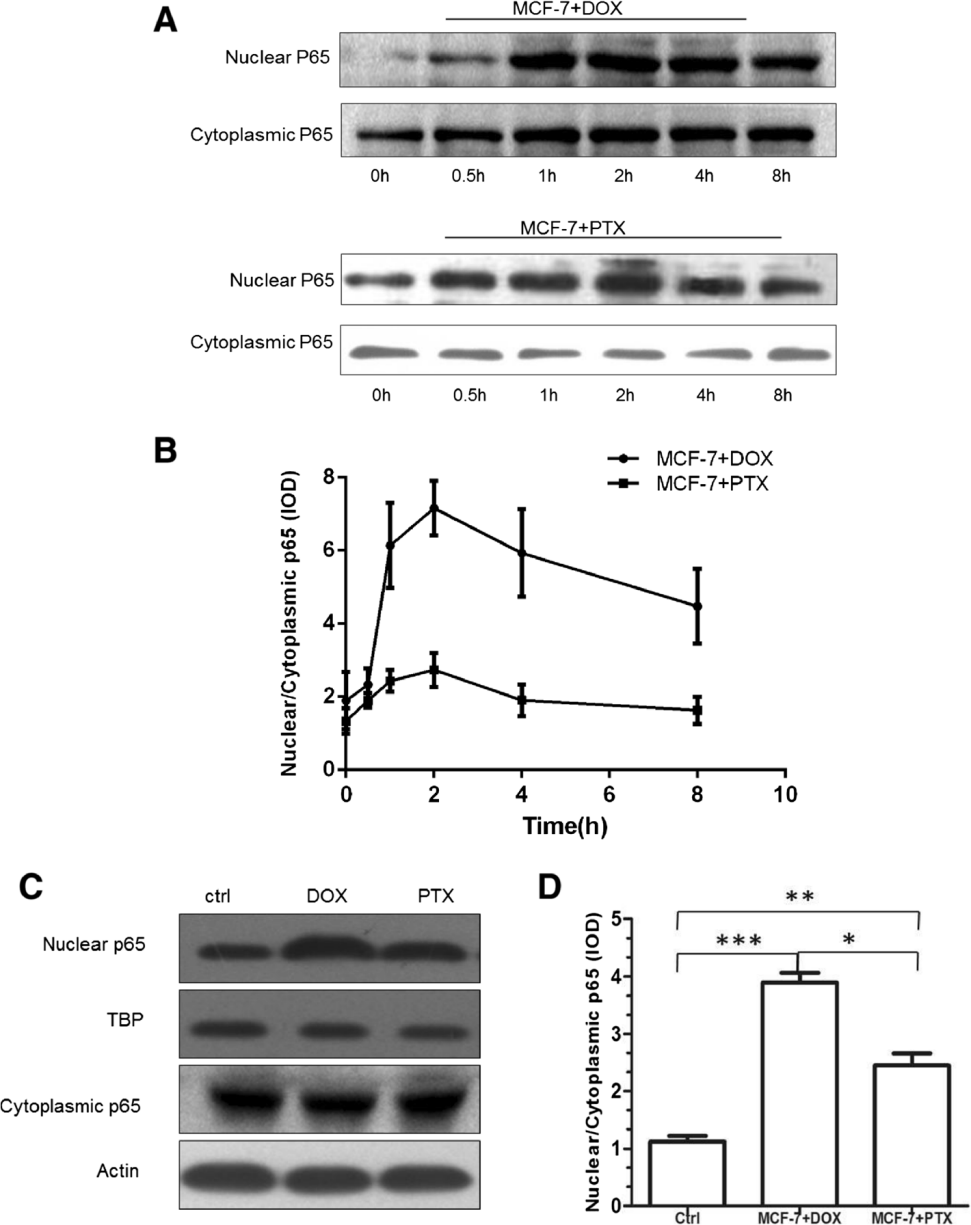
**p65 translocation in MCF-7 cells after the treatment of DOX and PTX.** **(A)** Western blotting and **(B)** nuclear:cytoplasmic ratio of NF-κB p65 in cytoplasmic and nuclear fractions of DOX and PTX treated MCF-7 cells at 0, 0.5, 1, 2, 4, 8 h. **(C)** Western blotting of NF-κB p65 after 2 h treatment of DOX and PTX using Actin and TBP as loading controls. **(D)** A quantitative analysis of nuclear:cytoplasmic ratio of p65 in **(C)**. *, *p* < 0.05; **, *p* < 0.01; ***, *p* < 0.001 (n = 3).

In terms of p65 translocation, Western blotting of cytoplasmic and nuclear p65 was performed. Actin and TBP were used as the loading control of cytoplasmic and nuclear fractions, respectively. As a result, both DOX and PTX enhanced the level of p65 in the nuclear region. Time-dependent analysis of NF-κB translocation from 0 to 8 h indicated a rapid translocation rate maximizing at 2 h (Figure 3A-B). In addition, a higher value was achieved upon exposure to DOX than PTX (Figure 3C-D). Of note, an apparent decline of p65 at time points longer than 2 h after the treatment of PTX was observed, probably attributed to the restored nuclear export of p65.

### Inhibition of NF-κB pathway

**Figure 4:**
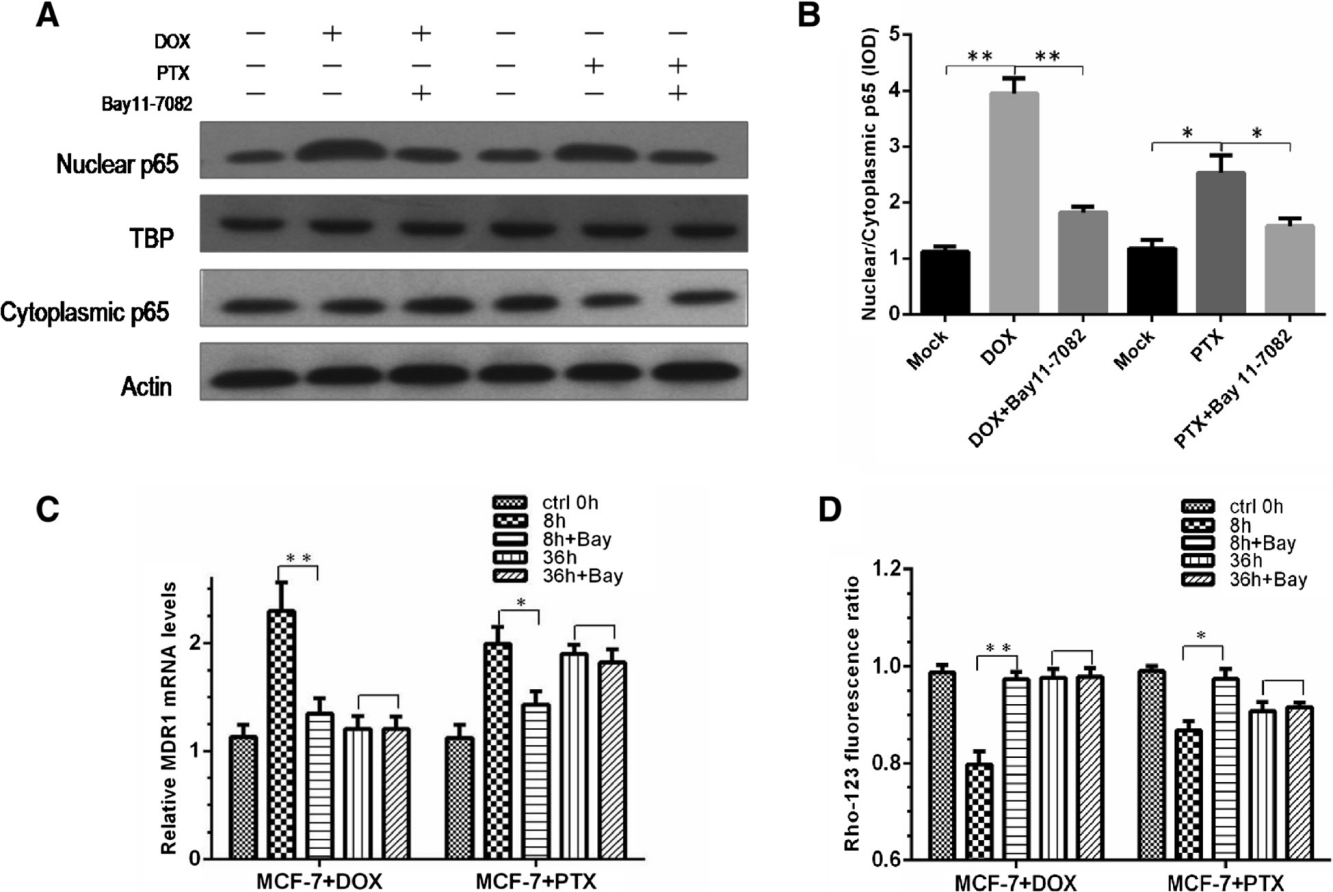
**Effect of NF-κB inhibitor Bay 11-7082 in DOX and PTX treated cells.** **(A-B)** Effect of Bay 11–7082 on the expression of NF-κB p65 in cytoplasmic and nuclear fractions with 8 h treatment of DOX and PTX and Bay 11–7082 for 2 h. **(C)** Relative MDR1 mRNA of DOX and PTX treated cells. *, *p* < 0.05; **, *p* < 0.01, blank *P* > 0.05 (n = 3). **(D)** Rho-123 fluorescence ratio of DOX and PTX treated cells. *, *p* < 0.05; **, *p* < 0.01, blank, *P* > 0.05 (n = 3).

To further investigate the NF-κB pathway, we examined the effect of Bay 11–7082 on P-gp expression and activity. Bay 11–7082 is an inhibitor of IκBα phosphorylation, thereby permitting IκBα to bind and inactivate NF-κB [17]. As shown in Figure 4A-B, both DOX and PTX displayed significantly lower entry of nuclear p65 in the presence of Bay 11–7082 at 2 h. The reduced mRNA expression and activity of P-gp were also observed (Figure 4C-D). In addition, P-gp returned to its baseline in DOX treated cells, whereas it only partially recovered in PTX treated cells and the P-gp induction was not completely abolished. Furthermore, inhibition of NF-κB by Bay 11–7082 at 36 h was not significant for both DOX and PTX treatments. Nevertheless, the resumed value at 36 h with the incubation of PTX was even greater than that at 8 h and of control, whereas DOX did not show such a trend, implying that at least another factor in addition to NF-κB participated in the P-gp modulation in PTX treated cells.

### NF-κB activation *via* TLR4

**Figure 5:**
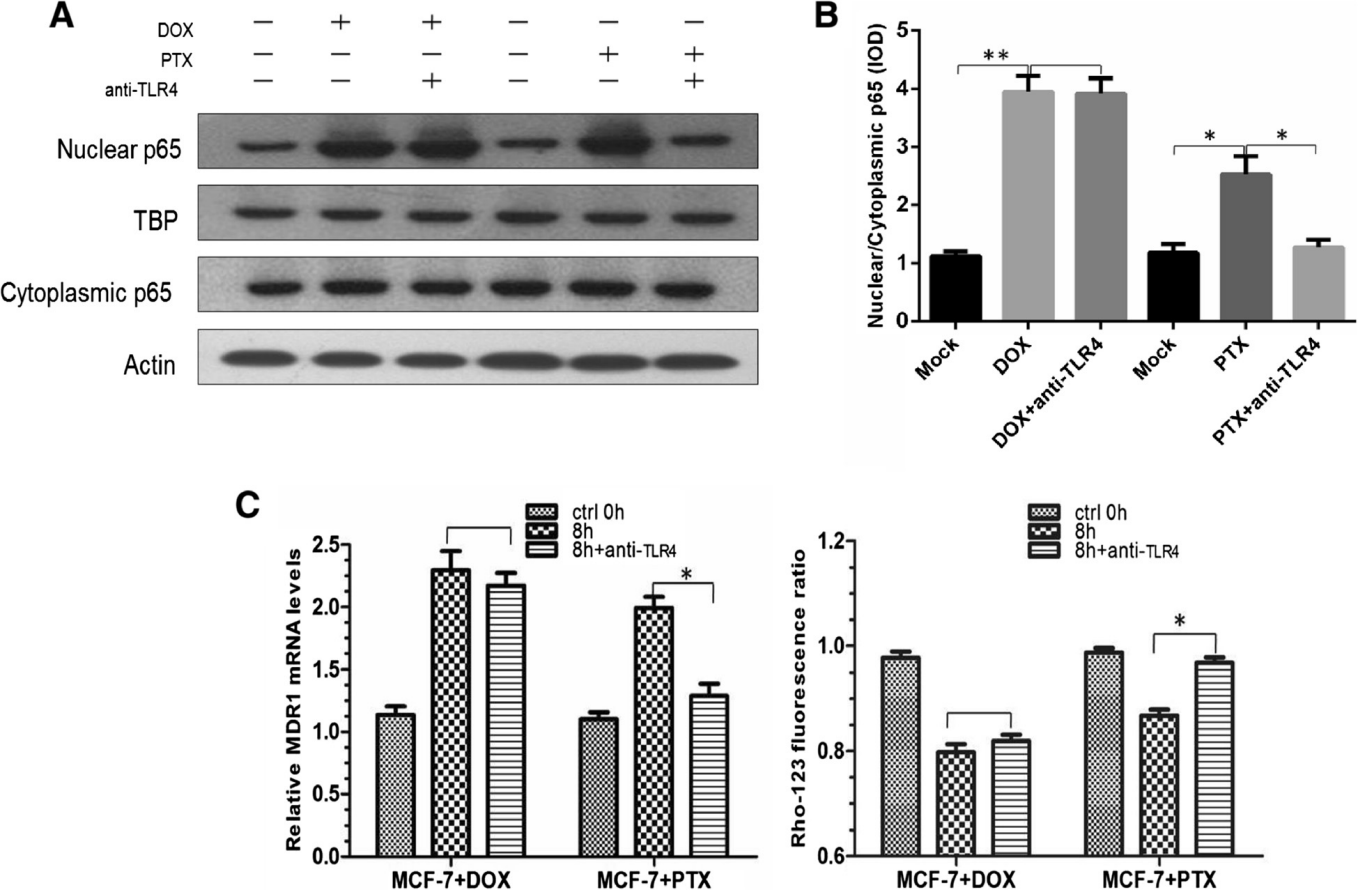
**Effect of anti-TLR4 antibody.** **(A-B)** Western blotting and quantitative analysis of nuclear and cytoplasmic p65 after the use of TLR4-blocking antibody. **(C)** Relative MDR1 mRNA level and Rho-123 fluorescence ratio of DOX and PTX treated cells. *, *p* < 0.05; **, *p* < 0.01, blank, *p* > 0.05 (n = 3).

In the upstream signal transduction of NF-κB activation, several signaling molecules can bind to their corresponding receptors, leading to the NF-κB signaling cascade [26]. Among them, TLRs generally signal through a MyD88-dependent manner, which involves the early activation of NF-κB [27]. As the first TLR discovered in humans, together with that both DOX and PTX have been reported as its potential ligands [28,29], TLR4 was examined in this study. The involvement of TLR4 was investigated using blocking antibody. The result indicated that PTX-induced NF-κB activation and P-gp overexpression were inhibited by anti-TLR4 antibody (Figure 5A-C). On the contrary, there was no significant alternation in P-gp level of DOX treated cells.

**Figure 6:**
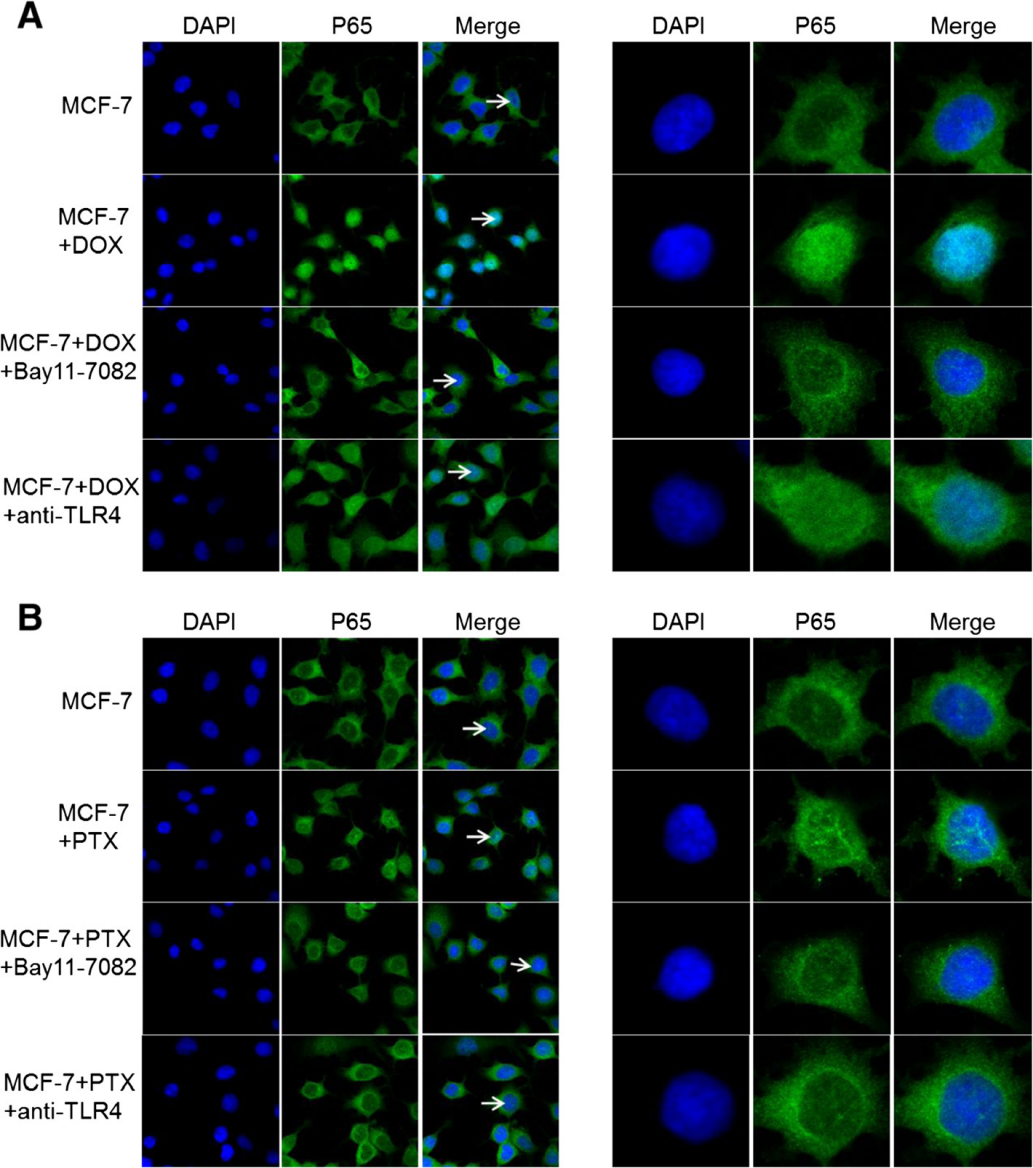
**Confocal images of p65 in cells after the treatment of DOX (A) and PTX (B) with or without Bay 11–7082 inhibition and TLR4 antibody blocking.** Cells were stained with Dylight 488 (green fluorescence) for p65 and co-stained with DAPI (blue) for positive identification of nuclei. The cells (arrow) are also enlarged at single-cell level for clarification (right images).

Finally, NF-κB pathway was also examined using confocal microscopy (Figure 6). The effects of anti-TLR4 antibody and Bay 11–7082 were demonstrated with Dylight 488 (green fluorescence) and DAPI (blue fluorescence) as p65 and nuclear stains. The obtained results were consistent with those of Western blotting. These data provided alternative evidence of NF-κB pathway underlying the drug resistance acquisition.

### Activation of PXR pathway

**Figure 7:**
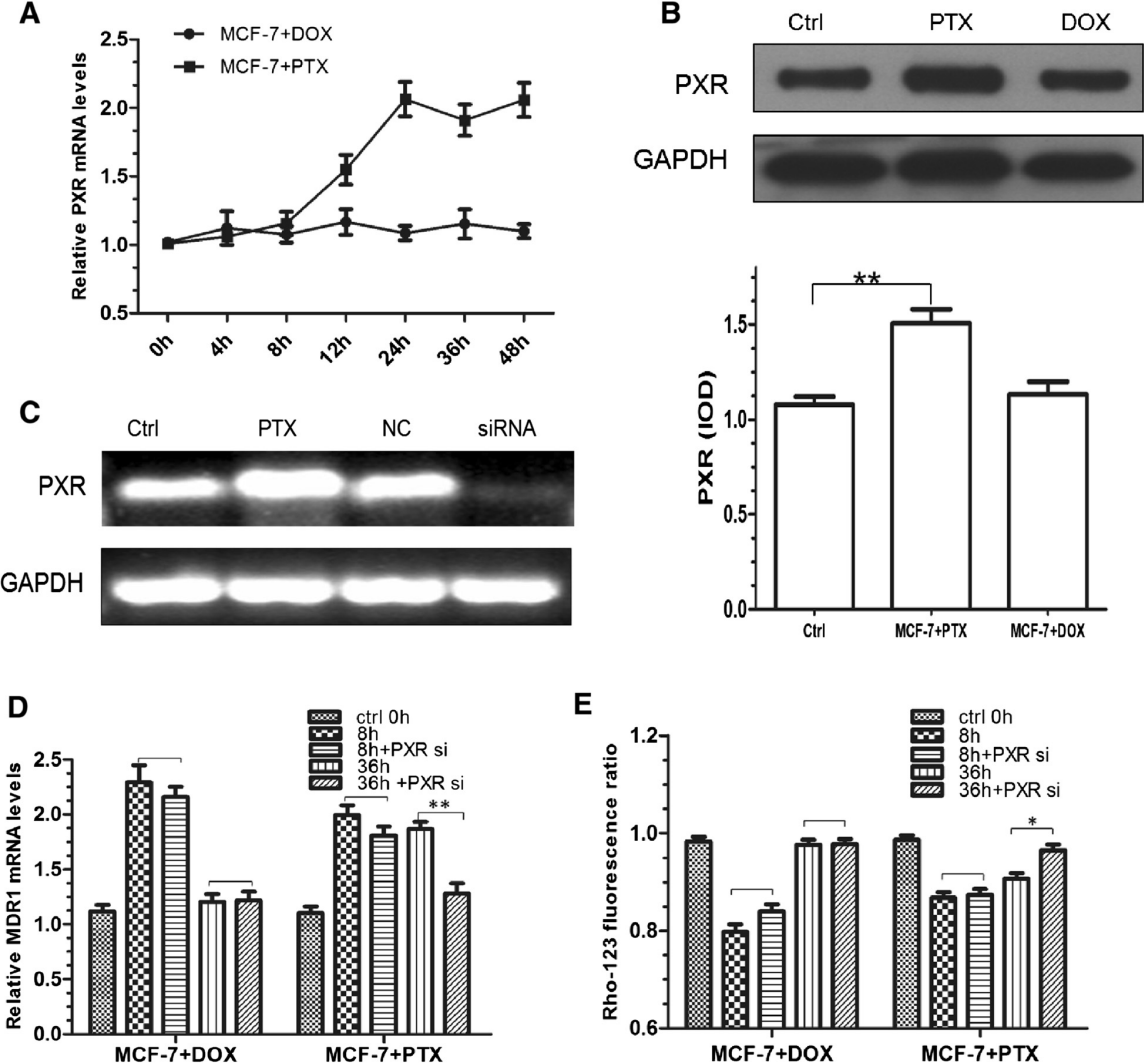
**Impact of PXR activation in DOX and PTX treated cells.** **(A)** PXR mRNA expression with the treatment of DOX and PTX at 0, 4, 8, 12, 24, 36, 48 h. **(B)** Western blot analysis of PXR with 24 h treatment of DOX and PTX. **, *p* < 0.01 (n = 3). **(C)** RT-PCR analysis of PXR mRNA in PTX only (PTX) or treated cells exposed to transfection conditions in the absence of siRNA (mock) or cells transfected with non-targeting (NC) or PXR-specific siRNA (siRNA). **(D)** Comparison of MDR1 mRNA expression in the cells exposed to transfection conditions with 8 h/36 h DOX and PTX treatment. **, p < 0.01 (n = 3). **(E)** Rho-123 fluorescence ratio in the cells exposed to transfection conditions with 8 h/36 h DOX and PTX treatment. *, p < 0.05 (n = 3).

Time-course study of PXR induction indicated that the expression of PXR mRNA increased with the treatment of PTX in the first 24 h (p < 0.05), and then kept on a plateau over 24 h later (Figure 7A). Correspondingly, PXR protein level enhanced in accordance with the observed level of mRNA (Figure 7B). On the contrary to PTX, DOX can not cause PXR change at both protein and mRNA levels.

To further examine whether the induction of DOX and PTX on P-gp was *via* the PXR activation, we designed a small interfering RNA targeting PXR to knock-down its expression. As a result, silencing efficiency of 80% was achieved compared with that of the non-targeting siRNA (Figure 7C). Neither mock (absence of siRNA) nor non-targeting siRNA had any effect on PXR mRNA levels. Then these silenced cells were used for PTX and DOX treatment and the result is shown in Figure 7D. While control cells (mock) exhibited an extent of P-gp induction by PTX, the siRNA-mediated PXR knock-down cells have an alleviated increase of P-gp in both expression and activity at 36 h (p < 0.01), strongly suggesting that this nuclear receptor was indeed implicated in the regulation of MDR1 gene. In contrast to PTX, DOX did not show such a result. There was no significant difference (p > 0.05) between the mock and siRNA transfected cells. Most importantly, relatively long-term activation of PXR was observed. To be specific, siRNA transfected cells had a significant decline of P-gp level at 36 h, but not at 8 h.

## Discussion

As earlier mentioned, DOX and PTX are used to treat breast cancer *via* distinct mechanisms [30]. The mechanism of DOX action is topoisomerase II inhibition and free radical generation. On the other hand, the effect of paclitaxel is related to its ability to bind to tubulin, to promote microtubule assembly, and to stabilize microtubules by bundle formation. In addition to this difference, there are several studies indicating that their ability to induce drug resistance is agent-specific [7,8]. However, few investigations provide significant insight into possible signal transduction pathways that enable breast cancer cells to acquire resistance to either PTX or DOX. In this study, MDR1 mRNA and Rho-123 assay were used to monitor the expression and activity of P-gp. The time-dependent result indicated that drug resistance induced upon exposure to PTX reserved after reaching the peak value, different from an apparent decline observed in DOX treated cells. This difference may be attributed to distinct ability of DOX and PTX to induce P-gp expression. It is reasonable to assume that multiple at least two pathways participated in the modulation of P-gp expression and function. One is short-term; the second one is relatively long-term.

There are several possible mechanisms underlying P-gp modulation disclosed over the past several years [31,32]. Among them, one potential intracellular target is NF-κB. As reported previously, both PTX and DOX shared the property of NF-κB activation [30]. Since transcription of IκBα is positively autoregulated by NF-κB, activation of NF-κB is usually self-terminated within minutes to hours, appearing short and transient [33,34]. In this study, the appearance of p65 at early time points (i.e., 2 h) and the incompetence of NF-κB inhibitor at a late time point (i.e., 36 h) were consistent with its rapid nature in *in vitro* studies [34]. Furthermore, the partially restored level of P-gp at 8 h and its enhanced level at 36 h in PTX treated cells in the presence of NF-κB inhibitor confirmed that the impact of NF-κB pathway was less significant on the resistance acquisition to PTX, and provided evidence that at least one more pathway took part in the PTX drug resistance acquisition than that of DOX.

PXR has been identified as another major regulator of P-gp induction [12,35]. Of the 170 xenobiotics tested, 54% of them demonstrated some level of PXR transactivation [13]. PTX was one of them. Different from PTX, DOX did not exert a significant effect on both PXR activation and the PXR-mediated induction of proteins, for example P450 3A4 (CYP3A4) [36]. Our results were in agreement with these findings, indicating that PTX but not DOX activated PXR-mediated P-gp induction and affected the expression and activity of P-gp. Compared to short-term activation of NF-κB signaling, the activation of PXR on P-gp seemed relatively long-term. The induction of PXR culminated at the time points (i.e., 24 h) longer than that of NF-κB and was then persistent at a stable level in our experimental period (i.e., 24–36 h). Similarly, the protein level of P-gp in PXR siRNA-transfected cells was more influenced at late time points with the treatment of PTX. Although this chronic effect has been previously reported *in vivo* [37,38], the direct comparison of the time dependence of NF-κB and PXR activation was firstly performed in this study. Notably, the combined data of these two pathways greatly matched the alternation trend of P-gp at protein and mRNA levels.

Despite of NF-κB and PXR signaling pathways investigated here, several issues observed in the experiments remained inclusive. For example, P-gp level was not equally induced by DOX and PTX *via* NF-κB activation. DOX treated cells obtained a higher P-gp expression and activity, which may lead to the ultimate higher P-gp level in MCF-7/DOX cultured cells. Unfortunately, the precise mechanisms that allow drugs to induce NF-κB activation have not been fully illustrated [39]. Since both DOX and PTX are potential ligands of TLR4 in the upstream signal transduction of NF-κB, we hypothesized here that TLR4 may be involved in the up-regulation of P-gp expression *via* NF-κB. The results indicated that there was a possibility that the acquired resistance to DOX was *via* or partially *via* NF-κB activation but not TLR4. PTX can induce the drug resistance *via* TLR4-NF-κB pathway.

It deserves to mention that TLR4 is not the single receptor upstream the NF-κB pathway. It was selected here largely due to it being as the most potential target of DOX and PTX. In addition to TLR4, NF-κB can also be activated by other receptors, such as IL-1R, TNF-R and other TLRs [26]. These upstream signaling components are also receptor-specific. Furthermore, the target gene products of NF-κB include many cytokines, chemokines and cell adhesion molecules [13]. Previous studies have shown that these products can be inducers of P-gp under several pathological conditions, such as infection, tumor, and diabetes mellitus [14-16]. For instance, DOX can inhibit the transcriptional activation of the oxidative stress-responsive heat shock factor 1 (HSF-1) and the subsequence declined expression of heat shock proteins in turn reverse the drug resistance by inhibiting MDR1/P-gp expression [40]. Such findings may be employed to explain drug resistance acquired upon exposure to DOX. Therefore, further studies will be required to address these issues.

Finally, we like to point out that the association between NF-κB and PXR signaling pathways was not explored in this study. In fact, recent studies revealed that a possible functional link may exist between them [41]. For instance, NF-κB/p65 can disrupt the connection of the PXR-RXRα complex with DNA sequences [42]. On the other hand, activated PXR was able to reduce the activity of NF-κB [43]. Thus, their mutual inhibitory potential deserves further assessment in the future.

Therefore, this study illustrated the differential drug resistance acquisition to DOX and PTX in MCF-7 cells by exploring NF-κB and PXR pathways. Recognition of this difference could help us to provide better treatment strategy in clinical practice. In the presence of other potential signaling components and interactions, more investigation is in demand.

## Conclusions

To our knowledge, this report is among the first to directly compare the time dependence of NF-κB and PXR pathways. Differential drug resistance acquisition to DOX and PTX in breast cancer MCF-7 cells was depicted. As a short-term activation, NF-κB participated in both DOX and PTX induction of P-gp. On the contrary, PXR pathway is a relatively prolonged activation and only PTX has an effect on the PXR-mediated induction of P-gp. This study provides significant insight into the possible pathways through which resistance to agents may be acquired in breast cancer. However, it deserves to point out that a wide variety of signaling pathways are involved in P-gp mediated MDR in addition to NF-κB and PXR, including mitogen-activated protein kinase (MAPK), c-Jun NH_2_-terminal kinase (JNK), p38, cyclic adenosine monophosphate-dependent protein kinase, phosphatidylinositol 3-kinase and protein kinase C [44]. Additional studies about these pathways will be required to better understand the drug acquisition process and provide strategies to circumvent MDR.

## Additional file

Additional file 1: Table S1. Sequences of Primers used for RT and Real-time PCR Analysis.
