# Supplementary material for: Differential drug resistance acquisition to doxorubicin and paclitaxel in breast cancer cells
Source: Cancer Cell Int. 2014 Dec 21;14:142. doi: 10.1186/s12935-014-0142-4 (PMC4279688; doi:10.1186/s12935-014-0142-4)
Supplement: Additional file 1: Table S1. — Sequences of Primers used for RT and Real-time PCR Analysis. [file 12935_2014_142_MOESM1_ESM.ppt]

## Slide 1
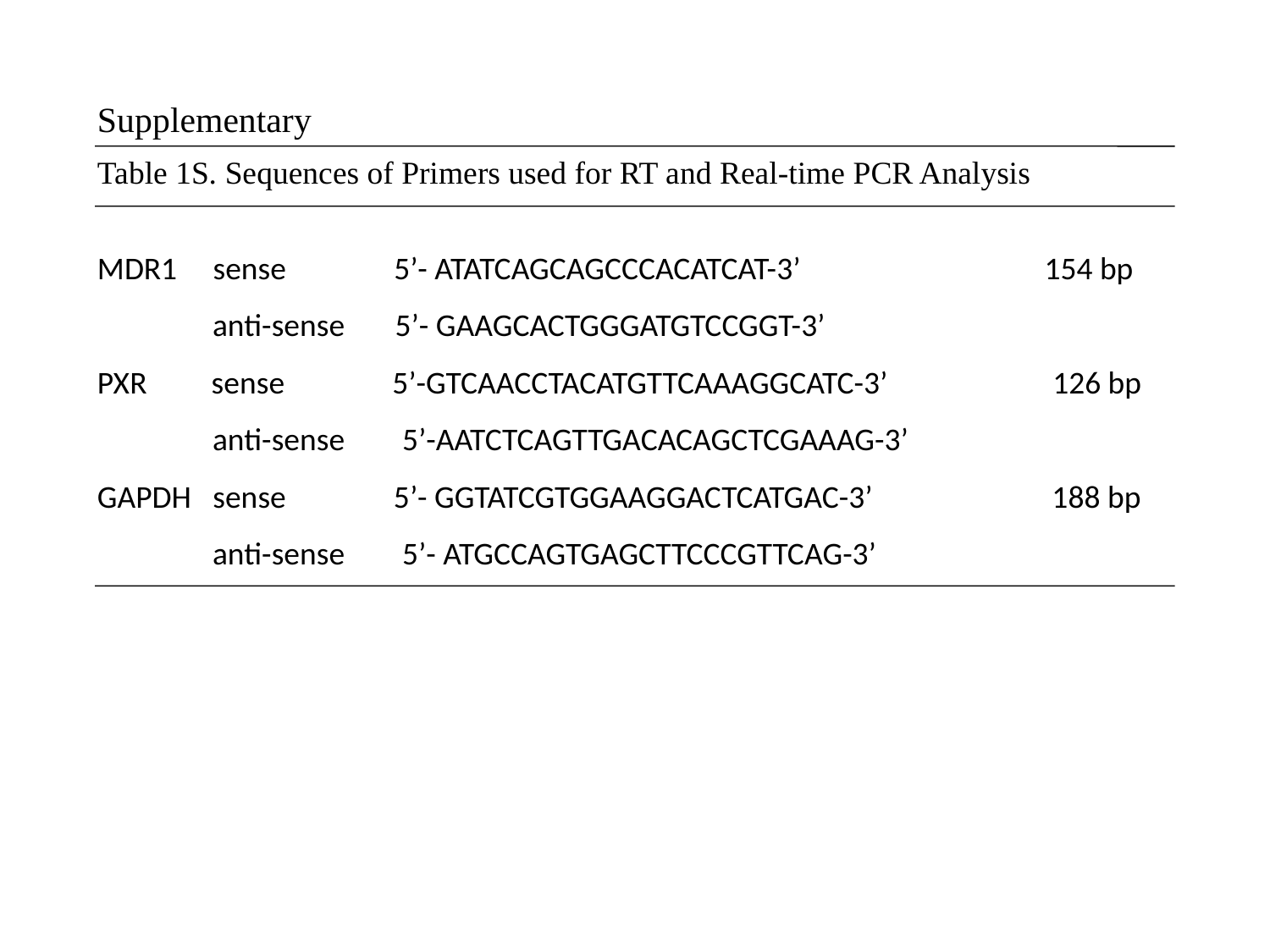

Supplementary
Table 1S. Sequences of Primers used for RT and Real-time PCR Analysis
MDR1 sense 5’- ATATCAGCAGCCCACATCAT-3’ 154 bp
 anti-sense 5’- GAAGCACTGGGATGTCCGGT-3’
PXR sense 5’-GTCAACCTACATGTTCAAAGGCATC-3’ 126 bp
 anti-sense 5’-AATCTCAGTTGACACAGCTCGAAAG-3’
GAPDH sense 5’- GGTATCGTGGAAGGACTCATGAC-3’ 188 bp
 anti-sense 5’- ATGCCAGTGAGCTTCCCGTTCAG-3’
